# The Rigidity of the Earth

**Published:** 1885-02

**Authors:** 


### THE RIGIDITY OF THE EARTH.

Sir Wm. Thompson recently delivered a lecture in Hopkins Hall, Baltimore, on "The Rigidity of the Earth." The object of the lecture was to show that the hypothesis to which the geologists have so rigidly clung, that the interior of the earth is continuously liquid, is not true. The theory is based upon the assumption that the deeper down we bore the warmer the temperature, until finally, at the depth of about 50 or 60 miles from the surface, the increase being constant and at the rate so far as observed, the temperature is far above the boiling point. This the lecturer characterized as a very large assumption upon very small premises. He endeavored to show that the hypothesis of those geologists who contended that the earth has been habitable for millions and millions of years is contrary to known physical laws, and then showed that the rigity of the earth, as demonstrated by the effect of the tides, could not be maintained if there was only a thin crust and a large liquid interior. The lecturer contended that the interior was not a continuous liquid mass, but probably consisted of mortared rocks, with limited spaces of liquid matter, so arranged as to support the exterior. Earthquakes he regarded as probably due to the falling in of solid matter, and their occurrence as evidence of this theory.

